# An Open‐Source, Three‐Dimensionally Printed, Motorized (“Breathing”) Nasotracheoscopy Simulator

**DOI:** 10.1002/oto2.70269

**Published:** 2026-06-19

**Authors:** Anthony M. Saad, David Herz, Mal Mehari, Andrey Filimonov, Kenneth Yan, Rachel Kaye

**Affiliations:** ^1^ Department of Otolaryngology–Head and Neck Surgery Rutgers New Jersey Medical School Newark New Jersey USA

**Keywords:** 3D printing, airway anatomy, flexible endoscopy, medical simulation, motorized larynx, nasotracheoscopy, otolaryngology education, simulation training

## Abstract

**Objective:**

To evaluate the face and content validity of a novel, 3D‐printed nasotracheoscopy simulator with a motorized, moving larynx using expert feedback from board‐certified otolaryngologists.

**Study Design:**

Device development and validation study.

**Setting:**

An academic institution.

**Methods:**

An anatomically accurate airway model was created from a de‐identified normal patient CT scan using open‐source segmentation software. Negative impressions of the turbinates and nasopharynx were 3D printed in PLA and cast in silicone for realistic soft tissue. A custom‐coded Arduino‐controlled servo motor was integrated to simulate laryngeal motion. Ten board‐certified otolaryngologists performed flexible nasotracheoscopy on the model and completed a survey assessing face validity, content validity, and general impressions using a 5‐point Likert scale. Descriptive statistics summarized responses.

**Results:**

The simulator received high scores for face validity (mean 4.6, SD 0.67), content validity (mean 4.8, SD 0.40), and general impressions (mean 4.83, SD 0.42). All participants agreed that the model accurately represented anatomical landmarks and held educational value. Feedback emphasized its utility for telescopic navigation and anatomical recognition training.

**Conclusions:**

This 3D‐printed simulator demonstrated strong validity and realism. Its anatomical accuracy, dynamic features, and reproducibility support its use as a training adjunct in procedural training. Future studies will assess its effect on learner performance.

Three‐dimensional (3D) printing has emerged as a promising approach to procedural training, offering an accessible, cost‐effective, and anatomically realistic alternative to cadaveric specimens and high‐end virtual platforms.^1^, ^2^ In otolaryngology, 3D‐printed simulators have been effectively used to teach complex procedures like sinus surgery, microlaryngeal surgery, and injection laryngoplasty, with multiple studies confirming their realism, educational value, and ability to differentiate skill levels.^2^, ^3^, ^4^, ^5^ These models have been shown using validated scoring tools to increase trainee confidence, improve technical performance, and facilitate structured feedback.

Flexible nasotracheoscopy is a technically demanding procedure that requires precise navigation through complex upper airway anatomy. It plays a critical role in the evaluation of upper airway obstruction, vocal fold mobility, and other laryngeal pathologies, making it an essential diagnostic tool in both emergent and outpatient settings. For trainees across multiple specialties, opportunities to develop these skills can be limited, often dependent on clinical volume, patient acuity, and available supervision.^6^ As the emphasis in medical education continues to shift toward structured, proficiency‐based learning, there is a need for accessible training tools that allow residents to build competence in a low‐risk, reproducible environment.^1^, ^7^

While simulation‐based training is gaining traction in airway management, few validated models exist for flexible nasotracheoscopy. The technique involves not only endoscopic navigation, but also real‐time spatial reasoning and a nuanced understanding of nasal, pharyngeal, and laryngeal landmarks, which are difficult to master through observation alone.^5^, ^8^ Nasotracheoscopy carries risks, including nasal cavity trauma, airway bleeding, and bronchospasm, underscoring the critical need for procedural proficiency before clinical application.^9^ Traditional training methods, such as mannequins or low‐fidelity task trainers, often lack anatomical accuracy or realistic tactile feedback, limiting their ability to translate into clinical performance.^2^, ^10^

To address this educational gap, we developed a 3D‐printed head and neck simulator specifically designed to replicate the nasotracheal pathway with anatomically accurate structures and a dynamic, motorized larynx. This simulator was designed to train and assess fundamental skills required for flexible nasolaryngotracheoscopy, including spatial orientation within the nasal cavity, bimanual scope manipulation, and anatomic landmark identification. These domains represent the core construct being evaluated and align with early trainee objectives in endoscopic airway education. Prior competency‐based frameworks in endoscopic sinus surgery have similarly identified spatial orientation, instrument handling, tissue respect, time and motion efficiency, and anatomic recognition as key predictors of operative performance.^11^ Establishing content validity for this simulator, therefore, required assessing whether its components and tasks adequately reflect these established educational goals.

This study evaluates the face and content validity of the model using structured feedback from board‐certified otolaryngologists. Expert participants performed flexible nasotracheoscopy using the simulator and completed a standardized questionnaire assessing anatomical realism, educational utility, and overall impressions. Our goal was to validate this novel simulator as a realistic and accessible tool for procedural training in airway endoscopy.

## Methods

### Simulator Design

A nasotracheoscopy simulator was developed to replicate the anatomical structure and dynamic motion of the upper airway, including vertical movement of the larynx. The simulator design was based on a de‐identified normal adult patient CT scan obtained from open‐source repository.^12^ Segmentation of the head and neck, nasal cavity, and the nasopharynx was performed using 3D Slicer (version 5.2.2; www.slicer.org), an open‐source medical imaging software.

**Figure 1:**
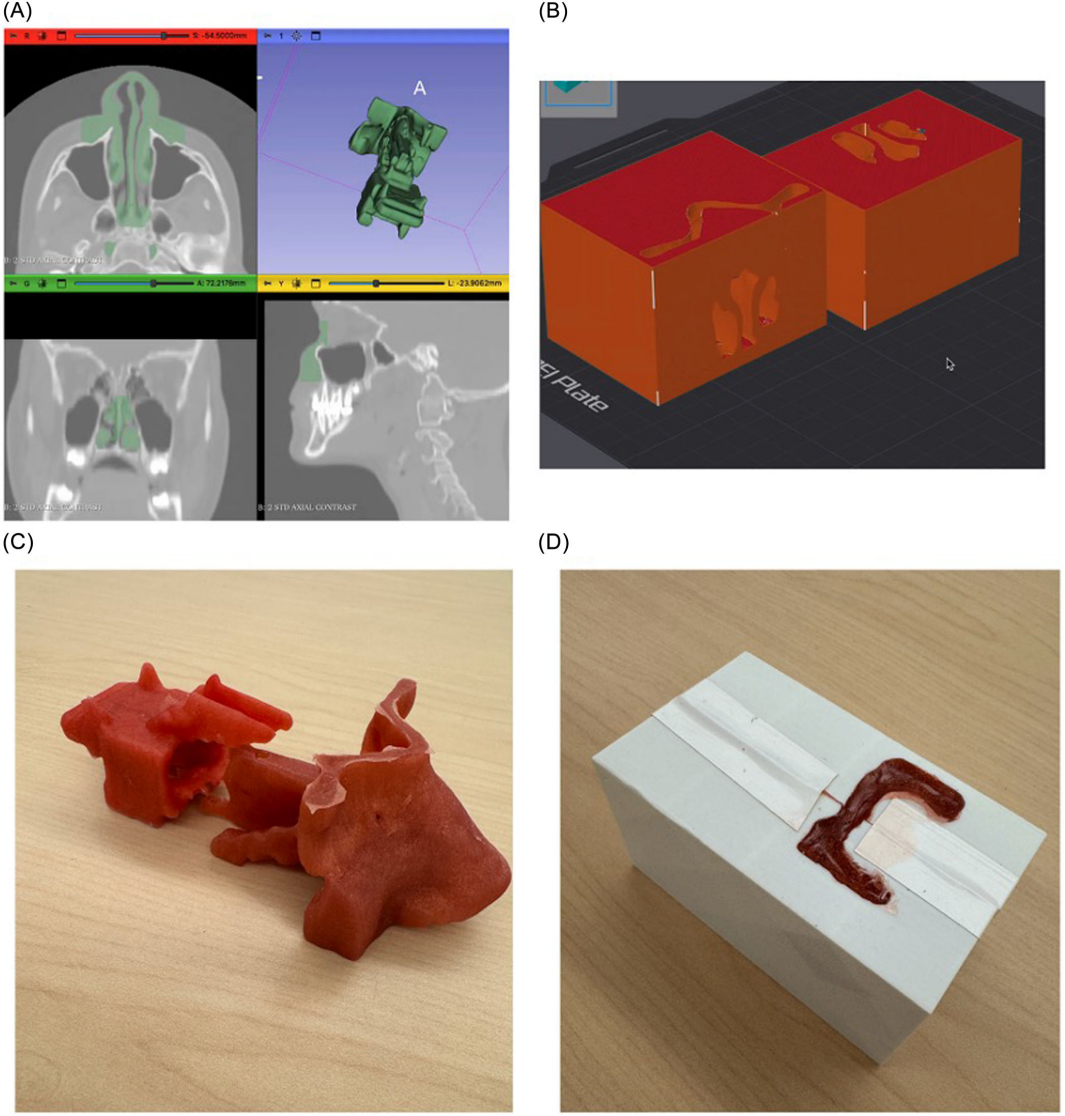
Process of soft tissue creation from left to right: (A) image of segmentation of turbinates in 3D‐Slicer program, (B) negative impression casts created using segmentation, (C) fully cured silicone inside 3D‐printed molds, (D) final product.

**Figure 2:**
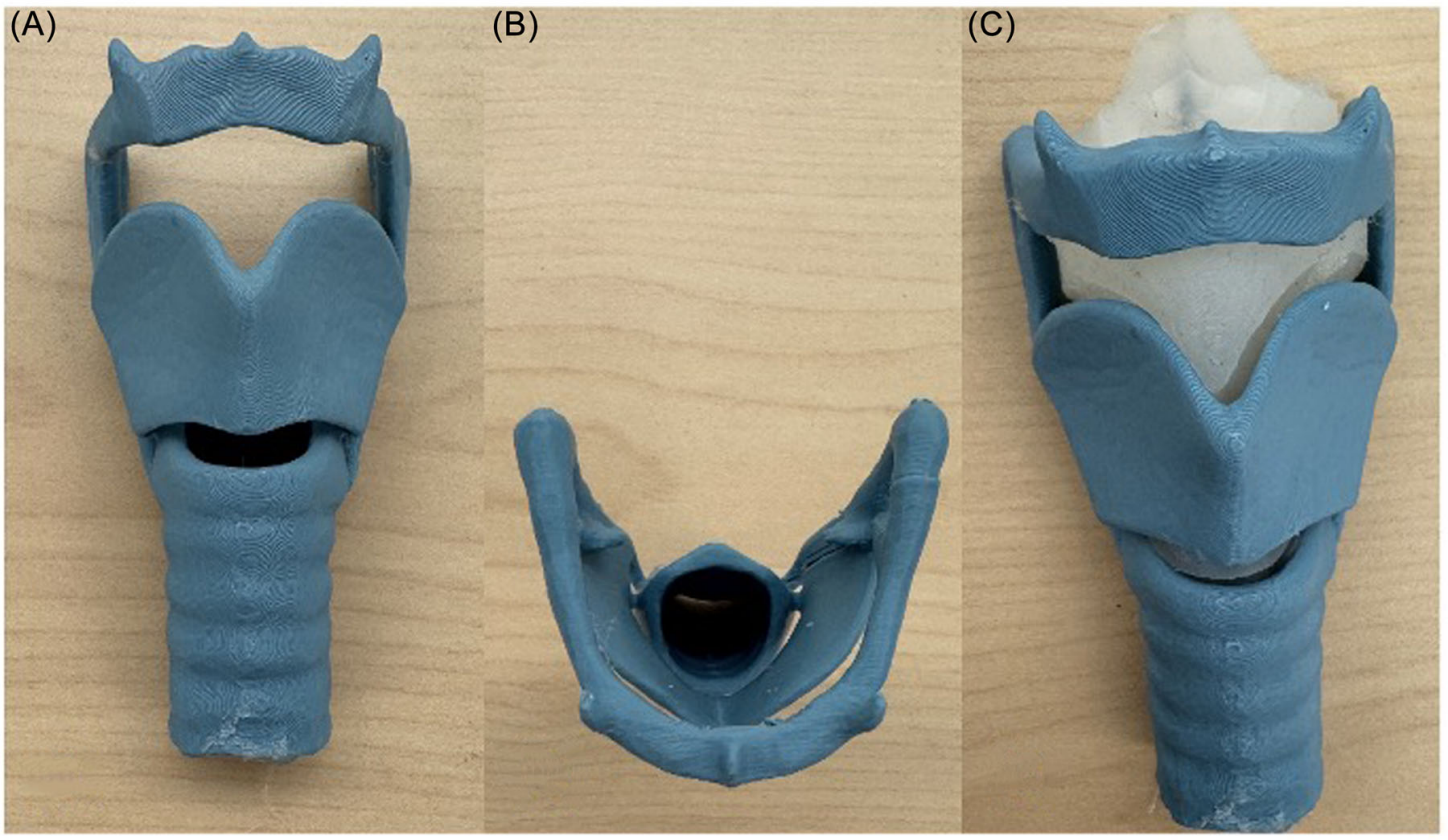
Rigid framework and compliant endoluminal components of the 3D printed laryngotracheal complex, from left to right: (A) anterior view of the rigid cartilaginous framework alone, (B) superior view down the laryngeal inlet, (C) anterior view of the assembled complex with the compliant endoluminal component seated within the rigid framework.

Negative impressions of the nasal cavity and turbinates were created by converting the segmented anatomy into stereolithography (STL) files and placing them into Bambu Studio (version 1.8.3). The impressions were printed on a Bambu Lab P1S 3D printer using standard 1.75 mm PLA filament. These negative molds were then cast with translucent, fast‐cure silicone rubber to produce flexible, realistic soft tissue structures for the nasal and nasopharyngeal regions including the turbinates and septum (Figure 1). An open‐source model for the hard and soft tissue of the larynx was printed to incorporate into the model^5^ (Figure 2). The model allowed full passage of a flexible nasoendoscope through the nasal cavity and into the larynx and trachea. All STL design files and assembly instructions are openly available at https://github.com/anthonymsaad/3D-Printed-Nasotracheoscopy-Simulator.

**Figure 3:**
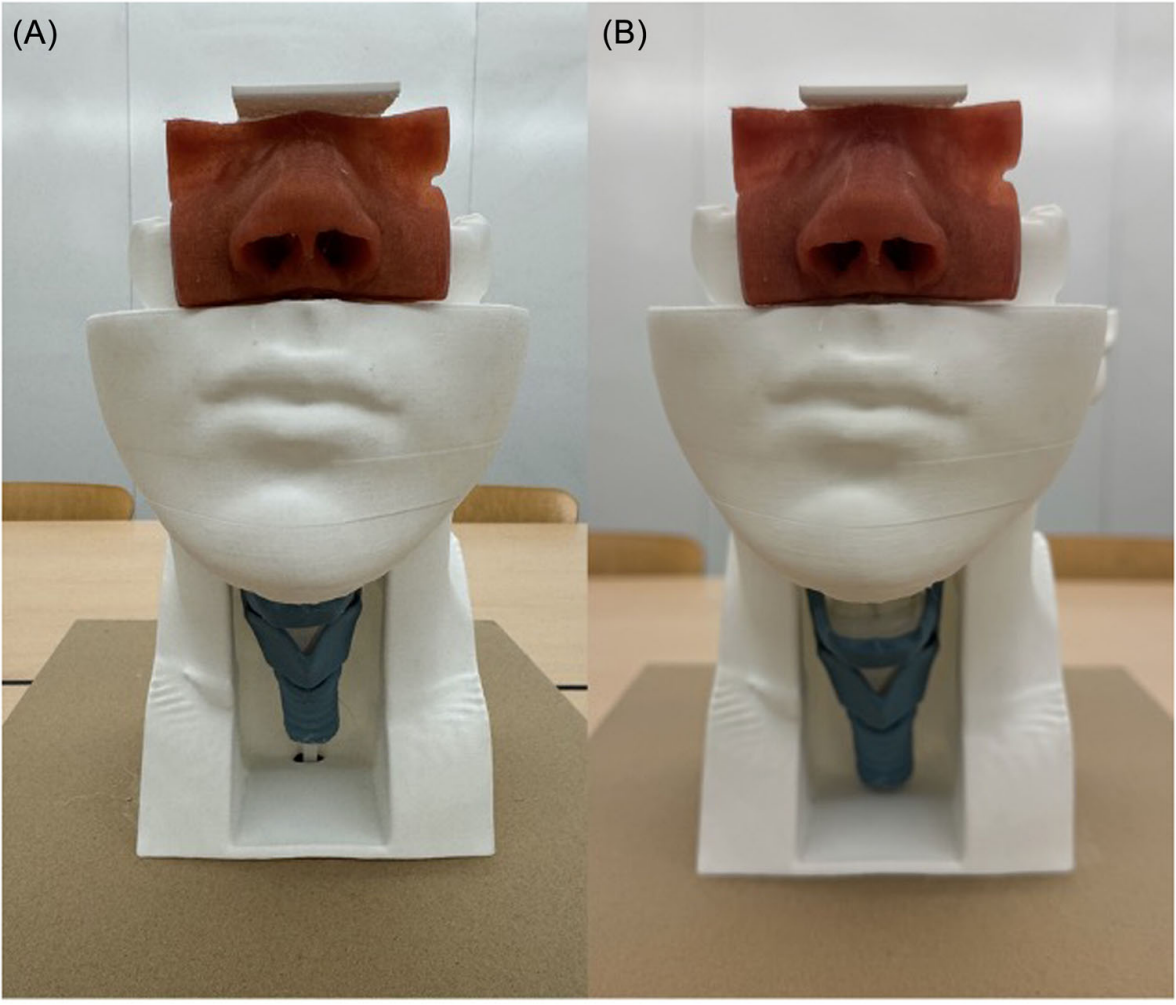
3D‐printed nasotracheoscopy simulator in superior (A) and inferior (B) laryngeal placement, showing oscillating capability of the larynx.

**Figure 4:**
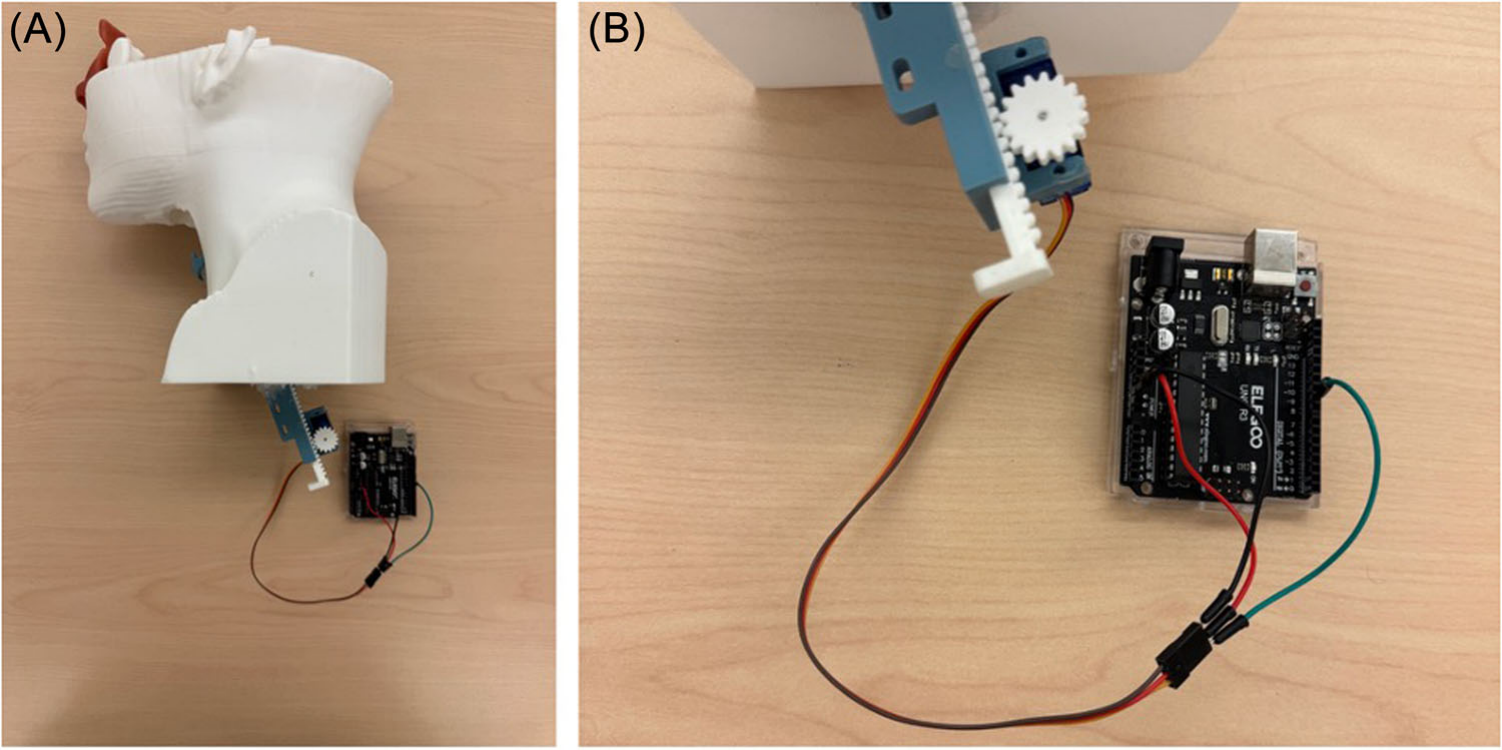
3D‐printed simulator on its side (A) showing the 3D‐printed linear‐rotary actuator system, along with a magnified image (B) showing the wiring of the servo motor to the Arduino board.

To simulate physiologic laryngeal motion, a servo motor (SG90) was embedded into the base of the model and programmed using an Arduino Uno microcontroller. The system was designed to move the entire larynx vertically in an oscillating pattern, approximately 2.5 cm in the superior and inferior direction, to mimic elevation and descent during breathing and swallowing (Figure 3). The motor was housed externally and connected to the laryngeal platform using a 3D‐printed linear‐rotary actuator system that allowed timed vertical motion during endoscopy (Figure 4).

The simulator underwent a rigorous development process consisting of 10 internal design iterations. Each version was evaluated by a fellowship‐trained laryngologist. Iterative changes were guided by qualitative feedback on anatomical accuracy, scope passage, tissue fidelity, and laryngeal motion. Design modifications included refinements to the curvature and width of the nasal passage molds, adjustment of soft tissue density through silicone casting parameters, and improvement of servo motor stability for more lifelike vertical laryngeal movement. This version of the simulator underwent one additional round of internal review by a second fellowship‐trained laryngologist and a fellowship‐trained rhinologist. These reviewers independently evaluated the model's anatomical realism, educational value, and overall quality. Each reviewer also performed flexible nasolaryngoscopy on the model and provided open‐ended comments to guide final optimizations. This multi‐phase, expert‐driven development process was designed to ensure anatomical accuracy of the simulator prior to formal validation testing.

The materials utilized to create this model consisted solely of the PLA filament and the silicone resin. With all parts, including the 3D‐printed molds used for the soft‐tissue, approximately 550 g of PLA ($0.017/g) was used. All together, without programming an Arduino Uno board to simulate movement, the cost of the static model was $25.54. A starter's kit that includes all of the wiring and material for programming and incorporation of the servo motor was purchased for $45.00, leading to a total cost of $70.54 for the dynamic model. The cost of the Bambu P1S 3D printer we used to print this model was approximately $699.

### Validity Assessment

Ten board‐certified otolaryngologists were recruited through academic and professional networks to evaluate the simulator. Participation took place in a controlled simulation setting at Rutgers New Jersey Medical School. Each participant performed flexible nasolaryngotracheoscopy using the simulator and was encouraged to assess anatomical realism, spatial orientation, and motion fidelity. Each faculty participant completed a single scope passage followed by an anonymous posttask survey assessing face validity, content validity, and overall impressions. All evaluations were performed using a 3.5 mm diameter flexible rhino‐laryngoscope which was visualized with a videomonitor. This scope was selected due to its widespread use in otolaryngology training and its compatibility with dynamic airway models.

Content validity was defined as the extent to which the simulator's anatomy and required maneuvers reflected the cognitive and psychomotor skills integral to flexible nasolaryngotracheoscopy. Participants rated anatomical realism, motion fidelity, and educational relevance, while face validity assessed overall realism and training utility. Because this pilot study relied on subjective ratings, subsequent evaluations will incorporate objective metrics such as task completion time and scope‐to‐mucosa contacts to establish construct validity, adapted from Balu et al. to maintain consistency across laryngoscopy simulation studies. Responses were recorded using a 5‐point Likert scale (1 = *strongly disagree*, 5 = *strongly agree*), with space provided for open‐ended feedback. This study was reviewed and approved by the Rutgers University Institutional Review Board (IRB# 00000610).

Descriptive statistics were used to summarize participant responses, including mean and standard deviation for each survey domain. All data were analyzed using Microsoft Excel (version 16.83). Statistical significance was set to *P* < .05.

## Results

A total of 10 board‐certified otolaryngologists participated in the simulator evaluation. All participants had completed residency training and reported regular use of flexible nasotracheoscopy in their clinical practice.

The simulator received high scores across all evaluated domains. The mean face validity score, reflecting perceived anatomical realism and fidelity of motion, was 4.6 (SD 0.67). Participants noted that the anatomical landmarks—including the nasal cavity, turbinates, nasopharynx, and larynx—were appropriately positioned and clearly identifiable (Table 1). The integration of dynamic vertical laryngeal movement added to the model's realism, per participant scoring.

**Table 1:** Face Validity, Average Score on a 5‐Point Likert‐Type Scale for Each Questionnaire Item (n = 10)

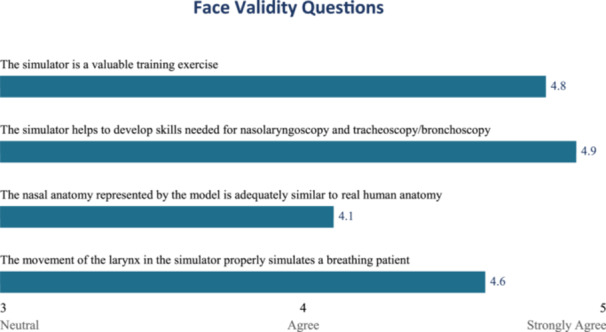

The content validity score, which assessed the simulator's value for teaching and training purposes, was 4.8 (SD 0.40). All participants agreed that the simulator had clear potential as an educational tool for training learners in flexible nasolaryngotracheoscopy, with unanimous strong agreement on helping develop bimanual dexterity and skills in flexible nasotracheoscopy (Table 2).

**Table 2:** Content Validity, Average Score on a 5‐Point Likert‐Type Scale for Each Questionnaire Item (n = 10)

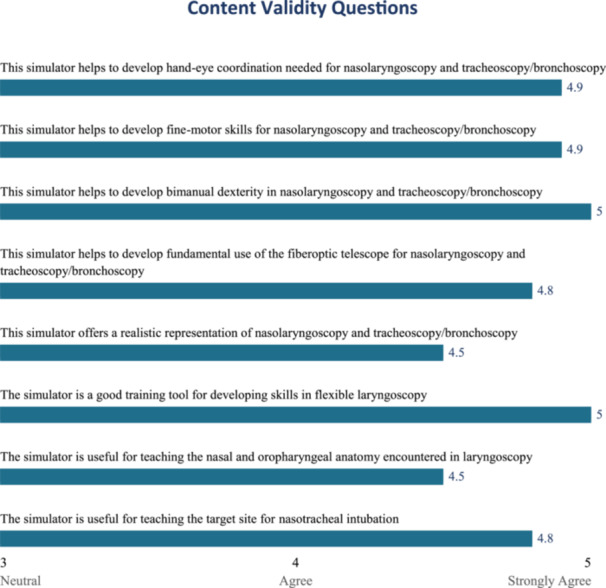

The general impression score averaged 4.83 (SD 0.42) (Table 3). Open‐ended feedback further supported the quantitative findings. Participants noted clear visualization of nasal and pharyngeal landmarks, with realistic passage of the scope through natural anatomical corridors. The oscillating vertical movement of the larynx was described as a “valuable addition” that helped simulate the dynamic conditions encountered during live examinations. Respondents suggested the simulator would be particularly useful in resident training, medical student education, and even for nonsurgical specialties such as anesthesiology residents. Several participants recommended minor refinements, including additional texture and color variation in the pharyngeal region and future incorporation of simulated secretions or variant anatomy to enhance realism for procedural skill development.

**Table 3:** General Impressions, Average Score on a 5‐Point Likert‐Type Scale for Each Questionnaire Item (n = 10)

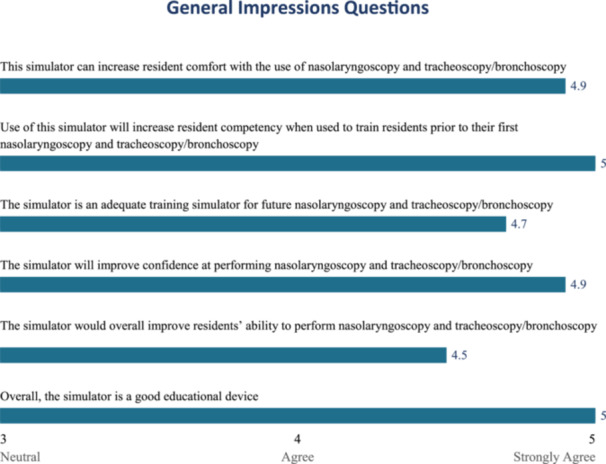

Our model can be accessed online on Wikifactory using the following link:

https://github.com/anthonymsaad/3D-Printed-Nasotracheoscopy-Simulator.

## Discussion

In our trials, all attending otolaryngologists agreed that the simulator could improve learner comfort, confidence, and competence in performing nasolaryngoscopy and tracheoscopy. This aligns with prior literature demonstrating that simulation, whether via low‐fidelity models or high‐resolution mannequins, can improve self‐assessed skill and comfort in nasotracheal intubation.^13^, ^14^, ^15^, ^16^, ^17^ For instance, a study involving emergency medical service providers showed increased nasotracheal intubation confidence scores following hands‐on training with a breathing mannequin, even among previously trained individuals.^18^ However, it is important to note the cost of this mannequin was over $2500, while our model costs $70.54 to produce. Even including the price of the 3D printer that was utilized at $699, our model still remains a lower‐cost option. Our findings support the model's utility not only as a training tool but as preparation for flexible endoscopy in airway management. By replicating the nasal‐to‐tracheal pathway along with the incorporation of a moving larynx, trainees are able to practice real‐time endoscopic control and spatial orientation.

Notably, attending physicians in our study agreed that the simulator realistically replicates nasolaryngoscopy and tracheoscopy. All confirmed that the nasal anatomy closely resembles human anatomy, supporting prior findings that anatomical fidelity enhances simulator value.^18^, ^19^ This is important as previous studies have shown that fiberoptic or blind nasotracheal intubation skills improve when realistic endoscopic views and tissue resistance are present.^20^ Attending evaluators strongly endorsed the simulator's effectiveness for teaching nasal and oropharyngeal anatomy, mirroring findings from Lee et al and Balu et al, who emphasized that anatomical realism and learner engagement enhance trainee confidence.^3^, ^5^

Previous 3D‐printed models have successfully simulated static procedures such as injection laryngoplasty and microlaryngeal surgery.^3^, ^5^ Prior models such as those by Gillespie et al and Lee et al, have been shown to be valid training tools for both novice and some experienced trainees.^5^, ^21^ Our model builds upon this foundation by simulating the complete nasal‐to‐tracheal pathway along with incorporating life‐like movement of anatomic structures. By incorporating anatomical realism and dynamic scope navigation, this simulator addresses a gap between prior models and the demands of airway management education. The integration of nasal, oropharyngeal, and tracheal components into a single modular platform represents a novel evolution in 3D‐printed airway training tools.

This model shows particular promise in potential utility as a training tool for teaching fiberoptic nasotracheal intubation. Nasotracheal intubation outcomes are influenced by a constellation of patient‐ and operator‐related factors, including body mass index, bleeding, airway secretions, and the number of prior intubations completed by the resident.^22^ Notably, even in the presence of complicating factors like nasal bleeding, success rates improve as residents gain cumulative experience, further highlighting the value of simulation‐based repetition in a controlled setting.^9^ Key procedural components—including scope control, anatomical recognition, and procedural sequencing—can be reliably acquired through simulation.^23^, ^24^, ^25^, ^26^ This evidence supports broader integration of simulation into airway management training, especially for procedures with steep learning curves or limited clinical exposure.^27^, ^28^

A unique feature of our simulator is its motorized larynx, allowing laryngeal elevation and movement during training—an innovation not found in previous models. This dynamic element simulates real clinical challenges during flexible endoscopy and nasotracheal intubation, where laryngeal motion can obscure the glottis or alter endotracheal tube trajectory. Currently published and available simulators are static and do not reproduce physiologic airway movement.^3^, ^5^ Ultrasound and videofluoroscopy studies have shown that during normal respiration and swallowing, the human larynx exhibits vertical excursions of approximately 1 to 1.5 cm during inspiration and up to 2.5 cm during swallowing, driven by coordinated muscular and skeletal motion of the hyoid and thyroid complex.^29^, ^30^, ^31^, ^32^, ^33^, ^34^, ^35^ These movements reflect essential biomechanical adaptations to airway control and protection. Even high‐fidelity animal models like Oberoi's 3D‐printed rabbit airway prioritized realism without dynamic features.^36^ While breathing mannequins have improved confidence in trainees, they lack active laryngeal movement and are often extremely costly.^18^, ^37^, ^38^ By integrating motion, our model introduces constructive difficulty and more accurately assesses readiness under lifelike conditions at a fraction of the cost.

Several limitations warrant acknowledgment. First, although the simulator provides detailed anatomical structures, the tactile properties differ from live tissue; PLA and silicone lack the same compliance of mucosa, similar to limitations seen in other models.^3^, ^5^ Second, the study was limited to faculty raters from a single institution. However, the number of total raters is similar to those published for other simulators. Additionally, while the motorized larynx enhances realism, it does not simulate complex physiologic behaviors such as vocal fold motion or a pharyngeal gag reflex. Finally, because all participants were attending otolaryngologists, future validation will incorporate novice learners, with blinded faculty assessors evaluating performance metrics such as time and scope contact frequency. Future studies should incorporate broader validation tools and comparisons with cadaveric or clinical performance to assess transfer validity.^39^, ^40^ Future efforts will focus on expanding simulator capabilities, including interchangeable anatomical variants (eg, septal deviation, turbinate hypertrophy, laryngeal edema), soft‐palate motion, external face‐like cover, and integration of motion tracking and OSATS‐based scoring to determine construct validity.^10^ We also plan to validate the simulator across multiple specialties and trainee levels.

## Conclusion

This study introduces a novel, low‐cost, 3D‐printed flexible nasotracheoscopy simulator with dynamic airway components. Highly rated by otolaryngology faculty for both anatomical realism and educational value, this simulator demonstrates strong potential for improving trainee preparedness for a variety of procedures, including nasotracheal intubation. By addressing a key gap in airway education, it offers a scalable, accessible tool for enhancing fiberoptic training in both anesthesia and otolaryngology.

## Author Contributions

**Anthony M. Saad**, conception/design, data acquisition/analysis, manuscript drafting, figure creation, final approval; **David Herz**, data acquisition/analysis, manuscript drafting, final approval; **Mal Mehari**, data acquisition/analysis, manuscript drafting, final approval; **Andrey Filimonov**, conception/design, data acquisition/analysis, manuscript drafting, final approval; **Kenneth Yan**, conception/design, data acquisition/analysis, manuscript drafting, final approval; **Rachel Kaye**, conception/design, data acquisition/analysis, manuscript drafting, final approval.

## Disclosures

### Competing interests

None.

### Funding source

None.

## Supporting information

Supporting Information.
